# Examining multiple funding flows to public healthcare providers in low- and middle-income countries — results from case studies in Burkina Faso, Kenya, Morocco, Nigeria, Tunisia and Vietnam

**DOI:** 10.1093/heapol/czad072

**Published:** 2023-11-16

**Authors:** Fahdi Dkhimi, Ayako Honda, Kara Hanson, Rahab Mbau, Obinna Onwujekwe, Hoang Thi Phuong, Inke Mathauer, El Houcine Akhnif, Imen Jaouadi, Joël Arthur Kiendrébéogo, Nkoli Ezumah, Evelyn Kabia, Edwine Barasa

**Affiliations:** Department of Health Systems Governance and Financing, World Health Organization, 20 Avenue Appia, Geneva 1211, Switzerland; Research Centre for Health Policy and Economics, Hitotsubashi Institute for Advanced Study, Hitotsubashi University, 2-1 Naka Kunitachi, Tokyo 186-8601, Japan; Department of Global Health and Development, London School of Hygiene and Tropical Medicine, 15-17 Tavistock Place, London WC1H 9SH, United Kingdom; Health Economics Research Unit, KEMRI-Wellcome Trust Research Programme, PO Box 43640-00100, Nairobi, Kenya; Health Policy Research Group, College of Medicine, University of Nigeria, Enugu Campus, Enugu 400001, Nigeria; Health Strategy and Policy Institute, Ministry of Health, 196 Alley, Ho Tung Mau, Cau Giay, Hanoi 100000, Vietnam; Department of Health Systems Governance and Financing, World Health Organization, 20 Avenue Appia, Geneva 1211, Switzerland; Morocco Country Office, World Health Organization, N3 Avenue Prince Sidi Mohamed, Suissi, Rabat 10000, Morocco; École Supérieure de Commerce de Tunis, Université de la Manouba, Tunis, Manouba 2010, Tunisia; Health Sciences Training and Research Unit, Department of Public Health, University Joseph Ki-Zerbo, 04 BP 8398, Ouagadougou 04, Burkina Faso; Health Policy Research Group, College of Medicine, University of Nigeria, Enugu Campus, Enugu 400001, Nigeria; Health Economics Research Unit, KEMRI-Wellcome Trust Research Programme, PO Box 43640-00100, Nairobi, Kenya; Center for Tropical Medicine and Global Health, Nuffield Department of Medicine, University of Oxford, Oxford 01540, United Kingdom

**Keywords:** Multiple funding flows, healthcare provider behaviour, strategic purchasing, healthcare financing, universal health coverage

## Abstract

Provider payment methods are traditionally examined by appraising the incentive signals inherent in individual payment mechanisms. However, mixed payment arrangements, which result in multiple funding flows from purchasers to providers, could be better understood by applying a systems approach that assesses the combined effects of multiple payment streams on healthcare providers. Guided by the framework developed by Barasa et al. (2021) (Barasa E, Mathauer I, Kabia E *et al.* 2021. How do healthcare providers respond to multiple funding flows? A conceptual framework and options to align them. *Health Policy and Planning*  **36**: 861–8.), this paper synthesizes the findings from six country case studies that examined multiple funding flows and describes the potential effect of multiple payment streams on healthcare provider behaviour in low- and middle-income countries. The qualitative findings from this study reveal the extent of undesirable provider behaviour occurring due to the receipt of multiple funding flows and explain how certain characteristics of funding flows can drive the occurrence of undesirable behaviours. Service and resource shifting occurred in most of the study countries; however, the occurrence of cost shifting was less evident. The perceived adequacy of payment rates was found to be the strongest driver of provider behaviour in the countries examined. The study results indicate that undesirable provider behaviours can have negative impacts on efficiency, equity and quality in healthcare service provision. Further empirical studies are required to add to the evidence on this link. In addition, future research could explore how governance arrangements can be used to coordinate multiple funding flows, mitigate unfavourable consequences and identify issues associated with the implementation of relevant governance measures.

Key messages

- Multiple funding flows from purchasers to providers can be better understood by applying a systems approach that assesses the combined effects of multiple payment streams on healthcare providers.
- While service and resource shifting occurred in most of the study countries, the occurrence of cost shifting was less evident.
- Among the attributes of the funding flows, the perceived adequacy of payment rates was found to most strongly drive change in provider behaviour.
- Because undesirable provider behaviour can negatively impact health system performance, future research should examine how governance arrangements can be used to coordinate multiple funding flows to mitigate unfavourable consequences.

## Introduction

Universal health coverage (UHC) is high on the global health policy agenda. Achieving UHC requires more than a simple increase in health spending; it also requires the efficient and equitable use of funds allocated to health (World Health Organization, 2010; Kutzin *et al.*, 2016; Cashin *et al.*, 2017). Recently, strategic purchasing, which deliberately introduces purchasing arrangements that encourage providers to pursue equity, efficiency and quality in service delivery (RESYST, 2014), has received increasing attention from researchers and policymakers (Cashin *et al.*, 2017).

Health systems in most low- and middle-income countries (LMICs) are financed using multiple sources, with funds channelled from health system purchasers to providers using a variety of payment arrangements and little, if any, central coordination (RESYST, 2016). Most analysis of provider payment focuses on the effects of individual payment mechanisms in isolation from other mechanisms. In 2017, a World Health Organization (WHO) global meeting on strategic purchasing concluded that mixed payment arrangements, which result in multiple funding flows from purchasers to providers, could be better understood by applying a systems approach to assess the combined effects, both complementary and antagonistic, of multiple payment streams on healthcare providers (WHO, 2017; Mathauer and Dkhimi, 2019).

Barasa *et al.* (2021) developed a conceptual framework for examining issues associated with multiple funding flows from the perspective of healthcare providers. The framework uses the term ‘funding flow’ to describe the transfer of pooled resources from a purchaser to a healthcare provider. A funding flow is characterized by distinct arrangements (attributes), such as services purchased, population group targeted, provider payment mechanisms, payment rates, accountability mechanisms and other contractual arrangements (Barasa *et al.*, 2021). While each funding flow has its own inherent incentives created by the arrangements made with providers (Cashin *et al.*, 2017b), multiple funding flows create a combination of the incentive signals sent by each separate funding flow.

Ideally, the incentives generated by each funding flow are complementary and compensatory to each other and create an overall blend of incentives that align provider behaviour with the objective of efficient, equitable and quality service provision (Barnum *et al.*, 1995; Langenbrunner *et al.*, 2009). However, without coordination and coherence, purchasing arrangements for individual funding flows are developed in isolation, and some incentives generated by the combination of multiple purchasing arrangements can neutralize, or even contradict, those of individual flows. When healthcare providers receive multiple funding flows, they may find certain funding flows more favourable than others, which may cause undesired provider behaviour that, in turn, could undermine the achievement of the health system objectives (Mathauer and Dkhimi, 2019). Barasa *et al.* (2021) produced an analytical framework that captures how multiple funding flows operate and how they are perceived by healthcare providers in order to further understand the combined effects of incentive signals created by multiple funding flows on healthcare provider behaviours. Guided by the framework, this paper synthesizes the findings from six country case studies in Africa and Asia that examined multiple funding flows and describes the potential effect on health provider behaviour.

## Methods

### Conceptual framework

**Figure 1.**
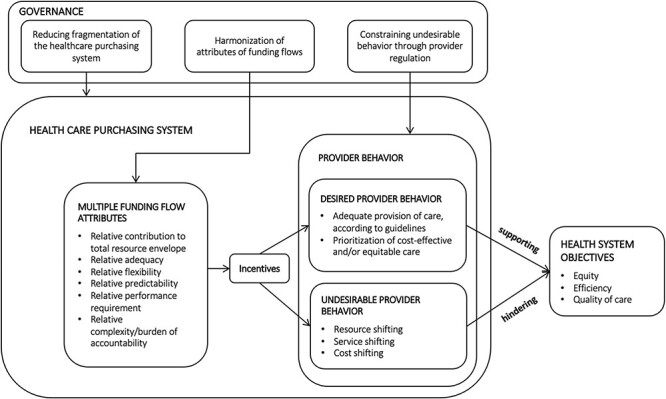
Conceptual framework of multiple funding flows

*Source:* Adapted from Barasa and Mathauer et al., 2021.

The study adopted the conceptual framework developed by Barasa *et al.* (2021) that hypothesizes that the presence of multiple funding flows and the associated attributes of each funding flow create a set of incentives that influence provider behaviour (Figure 1). The framework looks at the arrangements established between the purchasers and healthcare providers in each funding flow and identifies the incentives generated by the combination of funding flows by comparing the key attributes of each funding flow to determine how and why the mix of funding flows influences provider behaviour. The framework suggests that providers adjust their behaviour in response to the economic signals produced by multiple funding flows in a complex reaction, which occurs at both the individual (health personnel) and organizational levels. The behavioural response is driven by factors which may have consequences that are either positive, e.g. optimizing use of resources, improving quality of care, etc., or negative, e.g. delivery of unnecessary treatment, financial viability favoured over quality of care, resistance to change aimed at improving the use of resources, etc. While the range of potential healthcare provider behaviours in response to a set of multiple funding flows is extensive, the framework categorizes behaviour according to the potential pernicious effects on service provision (Barasa *et al.*, 2021), using the following categories:

- Resource shifting—which occurs when healthcare providers preferentially shift resources to provide services covered under a funding flow that is perceived to be favourable,
- Service shifting—which occurs when a provider shifts service provision from a funding flow considered to be less favourable to a funding flow considered more favourable and
- Cost shifting—which occurs when providers charge higher rates to some funding flows to compensate for lower rates from another funding flows.

This study focuses on the healthcare provider responses to the receipt of multiple funding flows, more specifically investigating the relationships between multiple funding flows and undesired provider behaviour. The framework can be further used to examine the effects of these behavioural responses on efficiency, equity and quality in service delivery; however, this dimension is not included in the scope of the present study.

### Methodology in the country case studies

The Resilient and Responsive Health System (RESYST) consortium (www.resyst.lshtm.ac.uk) undertook country case studies on multiple funding flows in Kenya, Nigeria and Vietnam in 2017. In parallel, WHO undertook country case studies in Burkina Faso, Morocco and Tunisia in 2017 and 2018. Table 1 summarizes the key features of the six country studies. The RESYST study was undertaken to better understand how multiple funding sources flow to healthcare providers and the likely implications of multiple funding flows on overall financing system coherence. The study focused on the issue of multiple funding flows in public healthcare facilities in selected geographical settings, and each study examined four public hospitals that receive funding from multiple funding sources (Mbau *et al.*, 2018; Oanh *et al.*, 2018; Onwujekwe *et al.*, 2018). The WHO studies were initiated as part of policy dialogue with governments planning to introduce strategic purchasing into their health systems The studies identified the healthcare service purchasers operating in the country, examined the payment arrangements used by purchasers and investigated any inefficiencies, inequities and quality concerns resulting from misaligned provider payment methods (Appaix *et al.*, 2017; Dkhimi *et al.*, 2017; WHO, 2020).

**Table 1.** Cross-country comparison in six countries

	RESYST studies			WHO studies		
Country	Kenya	Nigeria	Vietnam	Burkina Faso	Morocco	Tunisia
Data collection	Policy documentation review In-depth interviews Focus group discussion	Policy documentation review In-depth interviews Focus group discussion	Policy documentation review Review of facility data In-depth interviews Focus group discussion	Policy documentation review In-depth interviews	Policy documentation review In-depth interviews	Policy documentation review In-depth interviews
In-depth interviews	Total number: 36 Participants: County Department of Health officials, NHIF branch officials, doctors, clinical officers, pharmacists, nurses, hospital administrators, nursing officer in charge, medical superintendents	Total number: 66 Participants: State Ministry of Health, State Health Board, NHIS, Health Maintenance Organizations, doctors and nurses, hospital administrators	Total number: 10 Participants: Department of Health, Provincial Social Security office, District Social Security office	Total number: 67 Participants: Ministry of Health, SHI Fund, non-government organizations (NGOs) and community-based health insurance (CBHI) schemes running community health insurance, public and private hospitals	Total number: 32 Participants: Ministry of Health, Ministry of Finance, SHI Fund (Caisse Nationale de Sécurité Sociale (CNSS)), National Union of Mutual Insurance Agencies (Caisse Nationale des Organismes de Prévoyance Sociale (CNOPS)), district management teams, public and private hospitals	Total number: 17 Participants: Ministry of Health, Ministry of Finance; Ministry of Social Affairs, NHIF (Caisse Nationale d’Assurance Maladie (CNAM)), public and private hospitals
Focus group discussions	Total number: 4 Participants: service users	Total number: 8 Participants: service users	Total number: 12 Participants: doctors and nurses, service users	Not applicable	Total number: 1 Participants: regional hospital staff	Not applicable

The RESYST and WHO teams each developed their own study approaches, which they then shared at technical meetings and conferences. The teams reviewed each other’s study approaches with the viewing of synthesizing the study findings after the individual studies were complete. Although the RESYST and WHO country studies were undertaken separately, the aims and approaches to the studies were very similar in that the six case studies explored the funding flows from multiple purchasers to healthcare providers, examined the potential effects of multiple funding flows on healthcare provider behaviour and considered the impact of these behaviours on health system coherence and performance. The common aims and similarity in approaches ensured that the study findings were comparable. The RESYST and WHO teams held a face-to-face workshop in 2018 to discuss the findings in the respective studies with a view toward collating the findings from the analyses of different countries and synthesizing the empirical data collected in the studies.

Using a template developed based on the conceptual framework of Barasa et al. and Mathauer et al. (Barasa *et al.*, 2021), the country case studies were reviewed to extract findings on (a) the characteristics of the funding flows from all healthcare purchasers to the healthcare providers operating in a country; (b) evidence of resource shifting, service shifting and cost shifting behaviour in healthcare providers and (c) key attributes of the funding flows that can potentially explain healthcare provider behaviours. A cross-case synthesis, initially identifying within-case patterns and subsequently examining relationships repeated across both the WHO and RESYST case studies (Yin, 2018), was undertaken on the information collected in the template to determine: (a) the mix of funding flows received by healthcare providers and (b) the behaviours observed in healthcare providers and their perceptions of the key attributes of the multiple funding flows. The patterns identified in the template were further verified by the country study teams.

## Findings

### Description of the multiple funding flows

In all the study countries, the healthcare providers received funding flows from multiple sources and, in most cases, each purchaser used different payment arrangements to buy services from providers. Table 2 summarizes the funding flows identified in the study countries, providing an overview of the health financing system including (a) the financing mechanisms operating in the study countries, (b) the organizations that purchase healthcare services (purchasers), (c) the target populations for the financing mechanisms; (d) provider payment methods and (e) the services purchased by the provider payment methods. The target populations varied between financing mechanisms, but in some settings, a single financing mechanism targeted different populations using multiple funding pools, i.e. there were multiple funding flows within the one mechanism.

**Table 2.** Multiple funding flows in the case study countries

Healthcare financing mechanism	Purchaser	Target population	Provider payment method	Services covered
Burkina Faso				
(a) Tax-funded system	Central Ministry of Health (MoH)	Entire population	Line-item budget	Curative, preventive and promotive care
	MoH	Pregnant women, women of childbearing age, children under the age of 5 years	Fee-for-service	All services (except for chronic conditions) for children under the age of 5 years in the public facilities; for women: delivery, tests for cervical cancer, tests for breast cancer
	Municipal governments	Entire population	Line-item budget	Curative, preventive and promotive care
(b) Régime d’Assurance Maladie Universelle (Universal Health Insurance Scheme)	Caisse Nationale d’Assurance Maladie Universelle (Universal Health Insurance Fund—central) Pilot scheme in two districts	Pregnant women and children under age of 5 years	Case-based payments	All maternal services and all services for children under the age of 5 years in public facilities
(c) Community Health Insurance	NGOs and the network of CBHIs	Entire population	Fee-for-service	All services provided at the district level
			Case-base payments (in some instances)	
(4) Private health insurance	Insurance companies	Those covered by insurance	Fee-for-service Case-base payments (in some instances)	Services specified in the insurance contract
(e) Système de partage de coûts (cost sharing system—a solidarity fund supported by community contributions)	Health district (operated in a subset of districts)	Entire population	Fee-for-service	Specific services provided at the district level (e.g. emergency surgery)
(e) Result-based financing (RBF) scheme	The RBF national cell	Entire population in 19 districts (pilot scheme) from a total of 70 districts	Case-base payment (reward based on the number of services provided) Performance payment (reward based on attainment of specific quality targets)	Essential primary health care services delivered at the levels of government-owned health centres and district hospitals. Four modalities implemented: in some districts (Group 1), the scheme only rewards attainments in terms of volume of services and quality targets. In other districts (Group 2), it also includes payment of the exemption policy for the identified poor and vulnerable (‘the indigents’) for whom providers receive twice the amount to be normally paid under the fee schedule. In a third group of districts (Group 3), there is an additional performance reward attached to the number of identified poor and vulnerable patients seen by the medical personnel. Last modality, a combo of Financement Basé sur les Résultats as in Group 3 and CBHI offered to the whole population, is implemented in a fourth group of districts (Group 4).
(f) OOP payments	Individual households	Entire population	Fee-for-service	All services
Kenya				
(a) Tax-funded system	MoH	Entire population	Global budget	Inpatient and outpatient services; promotive, curative and rehabilitative care; palliative services provided by public tertiary and secondary county referral hospitals
	County Department of Health	Population within county	Line-item budget	Inpatient and outpatient services; promotive, preventive, curative and rehabilitative care provided by county public health facilities
(b) NHIF^a^	Civil servants’ contributions	Officers of the civil service in national and county governments	Fee-for-service	Dental care, optical care, Computed Tomography (CT) and Magnetic Resonance Imaging (MRI) scans for civil servants of all job groups; inpatient, outpatient, and maternity care for civil servants of higher job groups only
			Capitation	Outpatient services for civil servants of lower job groups
			Case-based payment	Maternity care for civil servants of lower job groups; renal dialysis, kidney transplant package, oncology package and surgical package for civil servants of all job groups
			Per diem	Inpatient services for civil servants of lower job groups
	Police and corrective service officers	Officers in the National Police Service and Kenya Prisons Service	Fee-for-service	Dental, optical care, CT and MRI scans for officers of all job groups; outpatient, inpatient services, maternity care for officers of higher job group
			Capitation	Outpatient services for officers of lower job groups
			Case-based payments	Maternal care for officers of lower job groups; renal dialysis, kidney transplant package, oncology package, surgical package for officers of all job groups
			Per diem	Inpatient services for officers of lower job groups
	National scheme	Any Kenyan of 18 years or older in the informal sector with no form of health insurance	Fee-for-service	MRI and CT scans; dental services
			Capitation	Outpatient services
			Case-based payments	Maternity care, renal dialysis, kidney transplant package, oncology package, surgical package
			Per diem	Inpatient services
	Health insurance subsidy programme for the poor	Indigent households or households with orphaned or vulnerable children, the elderly and persons with disabilities	Fee-for-service	MRI and CT scans, dental care
			Capitation	Outpatient services from contracted public or faith-based facilities
			Case-based payments	Maternity care, renal dialysis, kidney transplant package, oncology package, surgical package
			Per diem	Inpatient services
	Free maternity service scheme	Any pregnant woman without any form of health insurance who is a citizen of Kenya	Case-based payments	Maternity care at any public, faith-based or private facility
	County government schemes	Employees of county governments that have insurance with NHIF (14 out of the 47 counties)	Capitation or fee-for-service depending on the contract between the health facility and NHIF	Outpatient services
			Per diem or fee-for-service depending on the contract between the health facility and NHIF	Inpatient services
			Case-based payments	Maternity care, renal dialysis, kidney transplant package, oncology package, surgical package
			Fee-for-service	Dental and optical care; CT and MRI scans
	Schemes for parastatals and private organizations	Parastatals and private organizations that have insurance with NHIF	Capitation or fee-for-service depending on the contract between the health facility and NHIF	Outpatient services
			Per diem or fee-for-service depending on the contract between the health facility and NHIF	Inpatient services
			Case-based payments	Maternity care, renal dialysis, kidney transplant package, oncology package, surgical package
			Fee-for-service	Dental, optical care; CT and MRI scans
(c) CBHI scheme	NGOs, CBHIs	Principal contributor and declared dependants	Fee-for-service	Inpatient and outpatient care (chronic care often excluded) from contracted faith-based or private health facilities
(d) Private health insurance	Private health insurance funds	Private formal sector workers	Fee-for-service with co-payments Capitation	All services listed in the benefit entitlements, including inpatient and outpatient services, optical and dental care from contracted faith-based or private health facilities
(e) OOP payments	Individual households	Those without insurance coverage or those with insurance that require co-payments	Fee-for-service	All services, including promotive, preventive, curative, rehabilitative and palliative care
**Morocco**				
(a) Tax-funded system	Central MoH	Entire population	Line-item budget	Inpatient and outpatient services, chronic care, radiology, laboratory tests, drugs
	The scheme for the poor (Régime d’Assistance Médicale (RAMED))	Poor and vulnerable population (28% of the population)	Line-item budget	All services provided at hospitals and clinics
(b) SHI	Compulsory health insurance—SHI fund (CNSS)	Formal sector workers from the private sector	Fee-for-service with co-payments	Inpatient and outpatient services, chronic care, radiology, laboratory tests, drugs
	Compulsory health insurance—National Union of Mutual Insurance Agencies (CNOPS)	Formal sector workers from the public sector		
(c) Mutual insurance	Mutual insurance agencies	Formal sector workers	Fee-for-service with co-payments and ceilings	All services provided at public and private hospitals and clinics
(d) Private insurance	Private health insurance agencies	Private formal sector workers	Fee-for-service (with some ceilings and conditions)	All services provided at public and private hospitals and clinics
(e) OOP payments	Individual households	The uninsured	Fee-for-service	Inpatient and outpatient services, drugs, consumables (used in treatment)
Nigeria				
(a) Tax-funded system	Federal MoH	Entire population	Global budget	Preventive and curative services at the federal, state and local government levels
	State MoH		Line-item budget	Preventive and curative services at state and local government levels
(b) SHI (formal sector SHI programme)	NHIS	Formal sector workers (less than 5% of the population)	Capitation Fee-for-service	Primary (mostly curative) services Secondary and tertiary services
(c) CBHI	Communities and HMOs	Non-formal sector workers, voluntarily enrolled formal sector workers who are not covered by the Formal Sector SHI programme	Capitation and fee-for service depending on individual schemes	Mostly curative services
(d) OOP payments	Individual households	The uninsured	User fees	All types of services
Tunisia				
(a) Tax-funded system	MoH	Entire population	Line-item budget	All services at public health facilities
(b) SHI	National Health Insurance (CNAM)	Formal sector workers (public and private) and their dependants (68% of the population)	For the public sector: fee–for-service, up to an annual hospital ceiling (fees include medicines) with co-payments for vulnerable and CNAM affiliates	All curative medical services, including some high-cost items, drugs and medical consumables
			For the private sector: fee–for-service with co-payments (private and reimbursement affiliation)	All curative medical services, including some high-cost items, drugs and medical consumables
(c) Mutual insurance	Mutual insurance agencies	Formal sector workers	Fee-for-service (with ceilings and conditions)	All services in the private sector
(d) Private insurance	Private health insurance agencies	Formal sector workers	Fee-for-service (with ceilings and conditions)	All services in the private sector
(e) OOP payments	Individual households	Those not in prepayment schemes who are not insured and those paying for services above the insurance ceiling or for services not available in the public sector or services that are not included in the list of interventions covered by the CNAM	Fee-for-service	All services, including promotive, preventive, curative, rehabilitative and palliative care
Vietnam				
(a) Tax-funded system	Central MoH and local government (DoH)	Entire population	Global budget	Curative, preventive and promotive care
			Line-item budget	Preventive and promotive care
	National Target programme (MoH) (e.g. Tuberculosis control)	Entire population	Line-item budget	Activity based for the National Target programme (material inputs, incentive payments, information, education and communication activities, etc.)
(b) SHI	Vietnam Social Security (Central)	Entire population (86% enrolment in 2017)	Fee-for-service with ceilings	Curative services only (most diagnostic and therapeutic services that medical facilities provide and for which administrative user fees have been set; most drugs and medical consumables)
			Capitation, based on historical expenditure in the previous year in each province (pilot stage)	Outpatient services including referral to higher levels
(c) OOP payments	Individual households	Entire population (both non-insured and insured)	Fee-for-service at government set rates	Inpatient and outpatient curative services at all levels of healthcare facilities
			Co-payments for health insurance reimbursement	Benefit entitlements defined for health insurance
	Individual households (for ‘on-demand’ services)	Entire population (both non-insured and insured)	Fee-for-service at higher than government set rates	Inpatient and outpatient curative services at all levels of healthcare facilities

a Services provided under the NHIF in Kenya are provided by contracted, public, faith-based or private health facilities unless specified.

*Note:* The healthcare financing mechanisms are categorized as follows: (a) tax-funded; (b) mandatory health insurance; (c) private, not-for-profit health insurance; (d) private, for-profit health insurance; (e) other type of financing scheme and (f) OOP payments.

As indicated in Table 2, the number of funding flows received by a healthcare provider was determined by a combination of the number of healthcare financing mechanisms operating in a country, the number of funding pools in the financing mechanisms (e.g. sub-schemes for target populations and programmes for specific conditions/diseases) and the provider payment arrangements.

Of the study countries, Vietnam had the smallest number of financing mechanisms due to the fact that mandatory health insurance targets the entire population and is funded directly by government and out-of-pocket (OOP) payments, but has different provider payment arrangements that cover distinct service categories. In Kenya, a number of separate programmes operate under the National Health Insurance Fund (NHIF) to cover different sections of the population, which results in a large number of funding flows. There are also multiple financing mechanisms operating in the country. Numerous financing mechanisms operate in Burkina Faso, Morocco, Nigeria and Tunisia, where mandatory health insurance only covers a small proportion of the population, and there is a mix of both private non-profit and for-profit insurance mechanisms, as well as government schemes to provide priority services and support vulnerable populations. For example, in Morocco and Tunisia, in addition to the tax-funded system and the National Health Insurance Schemes (NHISs) covering the formal sector, large-scale medical assistance schemes that cover a large proportion of the population are funded through the government budget and complement free public primary healthcare centres to provide financial protection for the identified poor needing healthcare services.

### Changes in healthcare provider behaviour in response to multiple funding flows

#### Resource shifting

Resource shifting was found in nearly all the study countries (Table 3) wherein healthcare providers allocated more resources to the funding flows that they considered favourable. Typically, separate care pathways were created to allow more resources, including wards, staff, medical goods and equipment, to be given to patients covered by favourable funding flows. For example, in Kenya, providers dedicated specific wards and special clinics to patients enrolled in a scheme operated by the NHIF that covers civil servants. The dedicated wards and clinics were better resourced, in terms of staffing and healthcare commodities, than general wards. In Nigeria, hospitals established private laboratories that were better resourced than public laboratories to provide services for patients that paid by means other than through the public insurer, the NHIS. In Vietnam, hospitals collaborated with private industries to invest in more expensive and more modern equipment for ‘on-demand’ services, which were paid OOP. Patients receiving ‘on-demand’ services were treated in special wards, by higher skilled specialists and using better equipment than those were used for those receiving regular services that were paid using social health insurance (SHI) or user fees. Hospitals in Vietnam are not bound by standardized payment rates for on-demand services. Hospitals therefore charge higher prices for the services:

**Table 3.** Summary of the study findings—multiple funding flows and healthcare provider behaviour

	Multiple funding flows	Resource shifting	Service shifting	Cost shifting
Burkina Faso	Multiple financing mechanisms cover different population groups. Both not-for-profit and for-profit voluntary health insurance mechanisms operate in the health system. New healthcare financing schemes, funded by donor agencies, are being piloted, including SHI for maternal and child health. The tax-funded system charges user fees.	Human resources and time allocated to fulfil PBF reporting requirements. Health providers prioritize activities funded by PBF (e.g. health providers dedicate more time to outreach activities funded by PBF).	No examples identified	No examples identified
Kenya	A universalist health financing mechanism (NHIF) using multiple funding pools. A number of financing mechanisms exist, including not-for-profit and for-profit voluntary health insurance mechanisms. The tax-funded system charges user fees.	The civil servants’ scheme has special arrangements to deal with drug stockouts. Special civil servant clinics operate within public hospitals. ‘Amenity’ wards are available to NHIF beneficiaries (i.e. involving shifting staff, special equipment, additional beds).	Uninsured patients requiring long-term care or elective surgery are encouraged to enrol in NHIF.	No examples identified
Morocco	Multiple financing mechanisms cover different population groups. SHI targets formal sector workers. Both not-for-profit and for-profit voluntary health insurance mechanisms operate in the system. The tax-funded system charges user fees, except for those covered by ‘the Scheme for the Poor’.	Quicker attention given to patients covered by NHIS or by private insurance (e.g. shorter waiting times for appointments with specialists)	Patients covered by the medical assistance scheme are asked to purchase medicine from private pharmacies or pay OOP at public pharmacies.	No examples identified
**Nigeria**	Multiple financing mechanisms cover different population groups. Mandatory health insurance targets formal sector workers (5% of the population). Both not-for-profit and for-profit voluntary health insurance mechanisms operate in the system. The tax-funded system charges user fees.	Hospitals allocate more resources to the treatment of NHIS patients than fee-paying patients (e.g. dedicated doctors and consulting rooms).	Doctors prescribe medicine not included on the NHIS-approved drug list. NHIS clients are shifted from capitation to fee-for-service payments (i.e. from less profitable to more profitable funding flows). User fee clients are shifted from non-commercial (public-funded) to commercial (privately funded) laboratories in the hospital.	NHIS patients are charged higher rates than patients paying user fees for the same service (e.g. laboratory investigations).
Tunisia	Multiple financing mechanisms cover different population groups. SHI targets formal sector workers. Both not-for-profit and for-profit voluntary insurance mechanisms operate in the health system. The tax-funded system charges user fees.	No examples identified	Vulnerable patients who are covered by fee exemption schemes are often asked to purchase drugs from private pharmacies.	Private clinics sometimes charge patients insured with CNAM higher rates for the same service than they charge patients paying user fees.
Vietnam	A universalist health financing mechanism (SHI with 86% of the population enrolled) is supported by a number of smaller health financing mechanisms. A value-added ‘on-demand’ service charges user fee.	Hospitals invest in high-tech equipment and facilities for on-demand services and assign better qualified health workers to deliver on-demand services.	Patients with health insurance cards and user fee patients are shifted to on-demand areas if they can afford to pay. SHI patients are required to purchase drugs or other technical services from private facilities.	No examples identified

*There is an on-demand service area in the hospital, where specialist doctors, skilled doctors from leading central hospitals, are invited to come to provide patient care. There are 28 on-demand beds, and on-demand beds are available in all clinical faculties* (Provincial hospital manager, Vietnam).

The existence of a variety of payment rates for the same or similar services and the profitability associated with some payment rates appear to be the main drivers of resource shifting in the case study countries. Hospitals tend to dedicate more resources to areas where a higher income is expected. For example, healthcare providers consider the NHIF civil servants’ scheme in Kenya to be more favourable than the NHIF general scheme because it pays providers at higher rates. Public hospitals in Nigeria prefer payments to be made to their ‘private’ laboratories, and public hospitals in Vietnam prefer to supply ‘on-demand’ services because patients can be charged higher rates compared to the rates set by public health insurers in the respective countries.

Predictability in payments also seems to be an attribute that healthcare providers value. The regular transfer of payments under the capitation system used by the NHIF in Kenya was used by healthcare providers to justify a greater allocation of resources and preferential treatment of those covered by the NHIF:

*Capitation – you can predict how much you are going to get as a healthcare provider…* (Senior hospital manager, Kenya).

Performance-based financing (PBF) provides additional income to health facilities that achieve a target performance. As incentive payments for PBF aim to orient healthcare providers to deliver certain types of services and/or influence specific aspects of service delivery, PBF appears to drive a type of resource shifting behaviour in healthcare providers. For example, in Burkina Faso, health staff dedicated more time to outreach activities than other tasks if the outreach activities were paid through the PBF,

*Some activities, like home visits by some health centres, could reach 200 to 300 visits while in previous periods, there would be no more than 20 to 30 home visits. It was well paid because it received CFA 5000 [per visit from the PBF]* (District hospital manager, Burkina Faso).

#### Service shifting

In several case study countries, healthcare providers shifted services from a funding flow considered to be less favourable to a funding flow considered more favourable in order to maximize their own revenue (Table 3). A range of strategies were used to do this. In Kenya, providers encouraged uninsured patients requiring long-term care or elective surgery to enrol in the NHIF so that the providers could benefit from more secure and higher payment rates for the services than if the same patients had to pay the bill themselves:

*We usually encourage people to use [NHIF] cards because we consider them [NHIF patients] to be more important and it is actually even more important to the hospital when we have the cards, as we get higher returns…We usually prefer the NHIF cards but most of our patients don’t have them…Usually we do a lot of waivers and exemptions. Walk to our surgical ward, you can waive up to 100000 in a day. [A person has a bill of] 20000 but can only afford to pay 4000. We can’t keep that person in the ward but suppose now they had the cards… [then the cost of their treatment would be covered by the NHIF]* (Hospital accounts staff, Kenya).

In Vietnam, providers tended to encourage both SHI and user fee–paying patients to use on-demand services if the patients could afford to do so:

*Of course, there is a tendency for an extensive prescription of services for patients who pay user fees or use services on demand. This is for the convenience of both sides, and physicians can serve patients using better care…* (Provincial hospital doctor, Vietnam).

In Tunisia and Morocco, medical assistance schemes or fee exemption schemes remove the requirement of the poor and vulnerable from paying user fees at public healthcare facilities. However, the public health facilities are not compensated for the cost of delivering fee-exempted services and must cover the cost of the free healthcare services using their own budgets. As a result, public healthcare facilities often require exempted patients to buy medicines and other consumables at private pharmacies or undergo medical examination at private facilities and pay for these OOP payments. This type of behaviour is considered to be service shifting as healthcare providers move services from fee-exempted schemes to OOP payments in order to avoid a loss of revenue due to the fee exemptions (WHO, 2020).

A perceived inadequacy in payment rates is also a common driver of many of the service-shifting behaviours observed in healthcare providers—when the payment rate for one service is thought to be insufficient to cover the cost of providing that service, providers appear to transfer the service to other mechanisms to fund delivery. In Nigeria, case-based payments are used to purchase healthcare services for those covered by mandatory health insurance. However, due to a perceived low payment rate, providers ask patients to pay for the services OOP (Onwujekwe *et al.*, 2018).

In addition to the perception of payment rates by providers, the complexity of the accountability mechanisms associated with provider payments is an important factor in service shifting. In Vietnam, hospital managers noted that SHI requires hospitals to undertake a series of reporting and auditing activities, causing the workload for hospital administrative staff to increase (Oanh *et al.*, 2018).

On top of the increased workload caused by the mandatory reporting requirements of the health insurance scheme, provider claims for reimbursement can be rejected after auditing, which can be demotivating for healthcare providers:

*Hospitals are always under high risk of rejected reimbursement for any carelessness. For instance, in 2016, provincial general hospital X was denied reimbursement of claims worth VND 1-2 billion [USD 44000-88000] for the year. Moreover, patients with health insurance have more difficulties than fee-for-service patients in terms of long waiting times for the documentation of payment procedures* (Provincial hospital manager, Vietnam).

Increased workload resulting from accountability mechanisms can be an issue, particularly when hospitals suffer from scarce human resources. In Morocco, public hospitals favour budgetary allocations over payments involving billing and reimbursement processes because a lack of adequately trained administrative personnel and low compliance with reporting requirements by medical professionals make billing difficult resulting in services provided to SHI patients, which should be paid through fee-for-service by the SHI, being shifted to the hospital’s budget allocation:

*Often, when we send our bills to the National Health Insurance Scheme, the time-limit to submit them has already past, mainly because we lack administrative personnel to complete the claims, but also because doctors do not fill in the medical records as per requirements* (Public hospital accountant, Morocco).

#### Cost shifting

Different payment rates were often observed to be used for the same service under different funding flows. In Kenya, NHIF has higher payment rates for inpatient and specialized services (rebates, case-based payments, etc.) than rates applied under the user fee schedule:

*We have actually costed the surgical fees in this hospital. For minor surgery, in terms of time, resources, manpower, IV fluids, etc., it is about 5000 Shillings, while major surgery is about 10000 Shillings. NHIF gives [us] 30000 Shillings for minor surgery and 80000 for major surgery* (Senior-level hospital Manager, Kenya).

In Nigeria, fees for patients paying OOP are higher than those applied to mandatory health insurance members for the same service:

*Yes… for example, the hospital that normally does caesarean section for 150000 Naira (for OOP patients) but the NHIS tariff rate is 55000* (HMO representative, Nigeria).

While the difference in rates further explains why providers are tempted to encourage patients to be covered by funding flows with higher payment rates and to shift services to that funding flow, there is no clear evidence on whether the difference in payment rates occurred as a result of cost shifting and there is no clear indication that the price difference was used to cross-subsidize services offered under a lower paying scheme.

## Discussion

In many health systems in LMICs, more than one healthcare purchaser operates within the health system, which results in multiple funding flows reaching healthcare providers. Recent healthcare financing reforms seeking to progress towards UHC can also result in the creation of additional funding flows on top of those that already exist. Guided by the conceptual framework developed by Barasa *et al.* (2021), this study explored the extent to which numerous funding flows in multiple purchaser settings can affect healthcare provider behaviours in Burkina Faso, Kenya, Morocco, Nigeria, Tunisia and Vietnam.

This is the first study to systematically explore the potential for multiple funding flows in multiple purchaser settings in LMICs to affect healthcare provider behaviour. Healthcare purchasing issues associated with the existence of multiple payers have been examined in high-income settings, particularly in the USA where individuals are eligible for public and/or private health insurance and individuals can choose from a very large number of for-profit and not-for-profit insurance companies (Frogner *et al.*, 2011). Mcguire and Pauly (1991) modelled healthcare providers’ responses to payment rate changes in a multiple payer context and found that if physicians value maximum profit, when one payer reduces the payment rate for a service, there will be a reduction in the volume of that service and an increase in volume of services that do not have reduced payment rates, whereas if providers pursue a target income, they are likely to increase the volume of both services, even if one service has experienced a payment reduction. Tai-Seale *et al.* (1998) tested the McGuire and Pauly model with empirical data from the USA and showed that not all providers respond to payment reductions in the same way or in the way predicted by economic models. The authors argued that, in a multi-payer context, payment reductions by a single payer such as Medicare (a means-tested health and medical services programme for low-income households) are, at best, a partial solution to containing costs in the health system as providers respond to changes in payment methods or rates in various ways to align the changes with their own interests. While there is debate on cost shifting in the USA, where multiple private purchasers and Medicaid operate (Morrisey, 2003), empirical studies provide mixed evidence on the existence and size of cost shifting in US hospitals (Frakt, 2011), noting that cost shifting is often confused with price discrimination, where healthcare providers charge different purchasers different payment rates for the same services (Morrisey, 2014).

In this study, cost shifting occurred less frequently than resource shifting and service shifting. Cost shifting often occurs when highly autonomous providers negotiate payment rates with multiple purchasers (Barros and Olivella, 2011). Public providers with high levels of autonomy are less common in LMICs, which may explain why cost shifting behaviour was not often seen in this study. The case studies revealed that different payment rates are applied to the beneficiaries of mandatory health insurance and those paying user fees. In most of the study countries, no clear evidence was found that payment rates varied to subsidize the cost of providing healthcare services to those paying lower rates. Further investigation on the process of setting payment rates in the study countries is necessary to determine whether the price differences result from cost shifting, i.e. actor groups intentionally charging one purchaser higher rates to compensate for lower payment rates made by another purchaser.

Resource and service shifting was found to occur in most study countries. As suggested by the conceptual framework, these behaviours were incentivized by the attributes of multiple funding flows and can undermine the health system objectives of efficiency, equity and quality in healthcare service delivery. Although exploratory and qualitative in nature, the synthesis of the country experiences in this study revealed the risks for negative consequences for equity, efficiency and quality in healthcare service delivery due to the behaviour of healthcare providers receiving multiple funding flows. Further study, using both qualitative and quantitative approaches, will be useful to add to the body of evidence on the effects of resource shifting, service shifting and cost shifting on efficiency, equity and quality in service delivery in LMIC settings.

Of the attributes of funding flows considered in the analytical framework, the perceived adequacy of payment rates was reported to be the strongest driver of provider behaviour in multiple country settings. The predictability of payments and simplicity of accountability mechanisms are also important determinants of provider behaviour. These findings are consistent with previous literature reviews that showed that payment rates, predictability of payments and accountability mechanisms are the main determinants of provider behaviour (Kazungu *et al.*, 2018; Singh *et al.*, 2021). These are key elements to consider in the design and evaluation of payment methods as they determine the precise incentive(s) that a payment method sends to healthcare providers and may provide greater insight on how payment methods operate, beyond their descriptive label, e.g. fee-for-service, capitation, etc. For example, a performance reward does not trigger the same response from a provider when disbursement is delayed as when paid in a timely manner, as seen in Burkina Faso (Bodson *et al.*, 2018). In Nigeria, delays in payment to providers by the mandatory health insurance operators, together with provider dissatisfaction with payment rates, appear to have discouraged healthcare providers from treating members of the insurance programme (Etiaba *et al.*, 2018).

Policy responses are required to address concerns about the negative influence of healthcare provider behaviours resulting from multiple funding flows. Barasa *et al.* (2021) suggest that there are three broad approaches to the governance of healthcare purchasing in contexts where multiple funding flows occur: (a) reducing fragmentation in health financing to decrease the number of funding flows; (b) harmonizing the attributes of, and hence the signals sent by, multiple funding flows and (c) using legislative arrangements (e.g. the use of a regulatory framework) to constrain healthcare providers from responding in undesirable ways. Countries with a large number of financing mechanisms (such as Burkina Faso, Morocco and Nigeria) could expand the coverage of the publicly financed system (such as mandatory health insurance) and consolidate other financing mechanisms to reduce the number of funding flows and address issues associated with fragmentation. In countries where several programmes operate within a single financing mechanism, healthcare providers could receive multiple funding flows from that mechanism (such as NHIF in Kenya) and the coordination of purchasing arrangements should be considered by purchasers if each programme creates different purchasing arrangements with healthcare providers: standardization of purchasing arrangements across programmes would help to harmonize the incentives created by multiple funding flows. In countries where different types of funding flows exist (e.g. a large number of financing mechanisms and different payment arrangements between and within financing mechanisms), a combination of governance approaches (i.e. consolidation of funding mechanisms and standardization of purchasing arrangements) may be required. Several studies have provided further insights into how governance arrangements can improve the purchasing function, and a conceptual framework is available to assess purchasing governance arrangements (Mathauer *et al.*, 2019; World Health Organization, 2019; 2020; Organisation Mondiale de la Santé, 2020), but further empirical study is necessary to examine issues occurring in the process of managing multiple funding flows using governance approaches.

Health systems with multiple funding flows have a number of advantages, including the presence of alternative sources of funding for providers. The ‘resource shifting’ example of PBF provided in the Findings section can be an intended positive effect of multiple funding flows, encouraging more attention and resources to move to specific services and/or performance targets. However, balance needs to be found to avoid situations where providers shift resources to maximize income from PBF programmes at the expense of other service needs. The current study focuses on three behavioural changes by healthcare providers that result from the presence of multiple funding flows (i.e. resource shifting, service shifting and cost shifting), but the study lacks evidence that explicitly relates to the positive aspects of multiple funding flows. Thus, it is necessary to further investigate the benefits of multiple funding flows and articulate the benefits relative to the potential negative effects.

The findings from this study, mostly qualitative observations, reveal the potential for undesirable provider behaviours to occur as a result of the receipt of multiple funding flows and explain how certain characteristics of funding flows can drive the occurrence of such behaviours. Further investigation using robust quantitative evidence and/or mixed methods can deepen understanding of the links between multiple funding flows and the healthcare provider behaviours described in the analytical framework. Health system organization and institutional arrangements are equally important determinants of provider behaviour. Future studies should explore how different aspects of institutional and organizational environments, including the nature of healthcare purchasers, can influence the behaviours of healthcare providers operating under the context of multiple funding flows.

## Conclusion

This study reveals that undesirable provider behaviour can occur when providers receive multiple funding flows and explains how certain characteristics of funding flows can drive the occurrence of unwanted behaviours. To our knowledge, this is the first cross-country study to examine the links between multiple funding flows and healthcare provider behaviour in LMIC settings. Using the conceptual framework, countries wishing to further develop their purchasing mechanism could start by undertaking a detailed study of the multiple funding flows operating in their health system and describing the attributes of the funding flows and the effects on provider behaviour and, ultimately, on health system objectives, in order to understand the challenges and identify potential entry points for improvement. The country studies do not include a detailed examination of the effect of provider behaviours on equity, efficiency and quality, but indicate the negative consequences of the behaviours on health system performance. Further empirical studies are required to examine this link. In addition, future research could empirically explore how governance arrangements can improve the coordination of multiple funding flows to mitigate unfavourable consequences and identify issues associated with implementing suitable governance measures.

## Data Availability

The qualitative data underlying this article cannot be shared publicly to protect the privacy of the individuals that participated in the interviews and focus group discussions and to comply with the requirements of the ethics approval.

## Reflexivity statement

Among the authors, there are eight females and five males and a range of levels of seniority are represented. Of the 13 authors, nine are from the global south: five from sub-Saharan Africa, three from North Africa and one from Asia. Two authors have medical backgrounds, but all the authors have been trained in either health economics or sociology. All the authors have extensive research experience in health financing in LMICs and are knowledgeable in the application of qualitative research methods in health systems and policy research.
